# Supplementary material for: Treating coral bleaching as weather: a framework to validate and optimize prediction skill
Source: PeerJ. 2020 Jul 3;8:e9449. doi: 10.7717/peerj.9449 (PMC7337031; doi:10.7717/peerj.9449)
Supplement: Supplemental Information 1 [file peerj-08-9449-s001.pdf]

Supplement: Figures S1, S2, and corrections made to the Hughes et al. (2018) bleaching database.

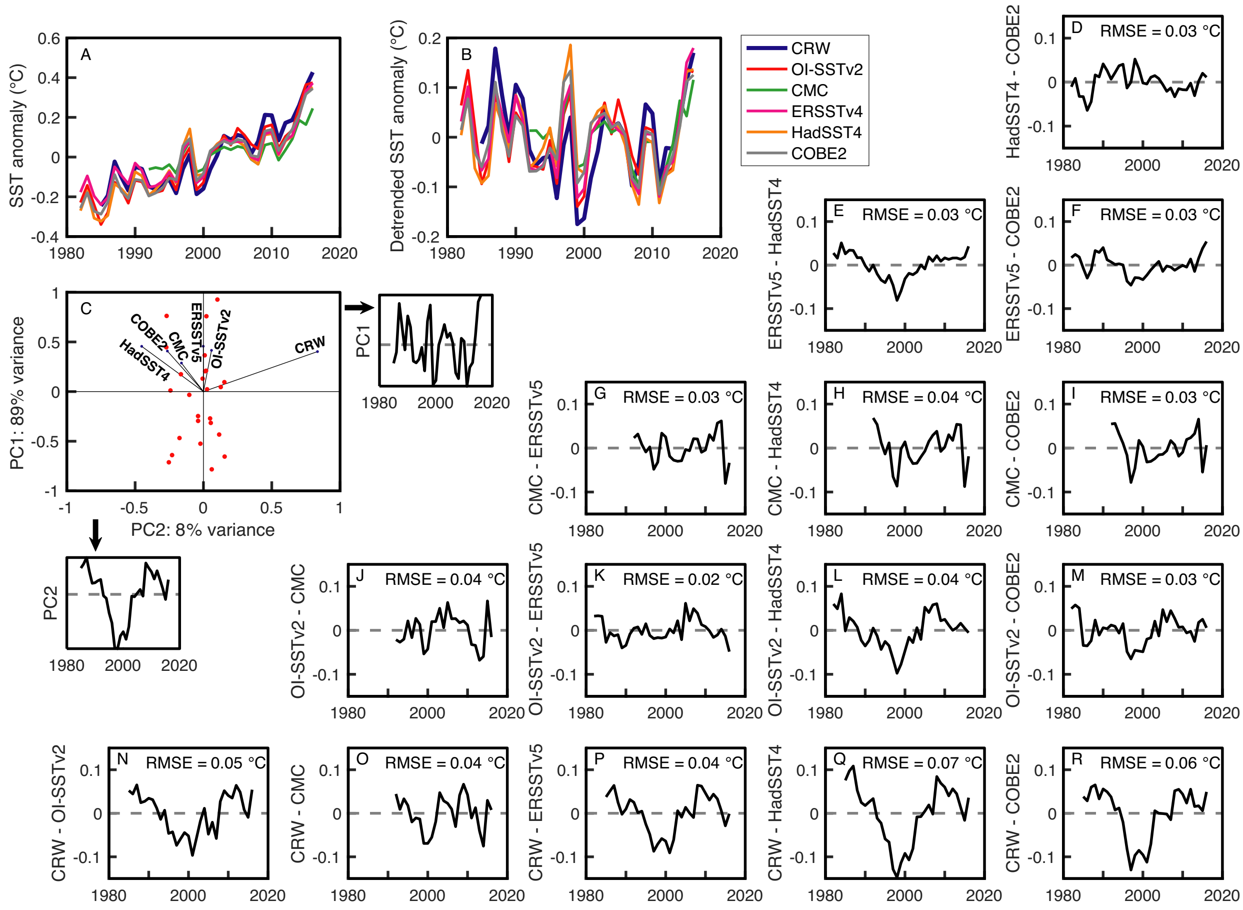

Figure S1. Comparison of global-mean annual sea surface temperature (SST) anomalies among various SST products. (A) Global-mean SST anomalies from 1982-2016, and (B) the same data but detrended. (C) Principal component analysis (PCA) of the detrended annual SST anomalies, with small side panels showing the time series of PC1 and PC2. (D-R) Pairwise differences of the global-mean anomalies between SST products. Root-mean-square error (RMSE) in each panel indicates the degree of mismatch between each pair. Note that PC1 (plotted from 1985-2016) largely reflects the detrended global-mean time series (B), whereas PC2 is similar to the mismatches between Coral Reef Watch and other products (N-R).

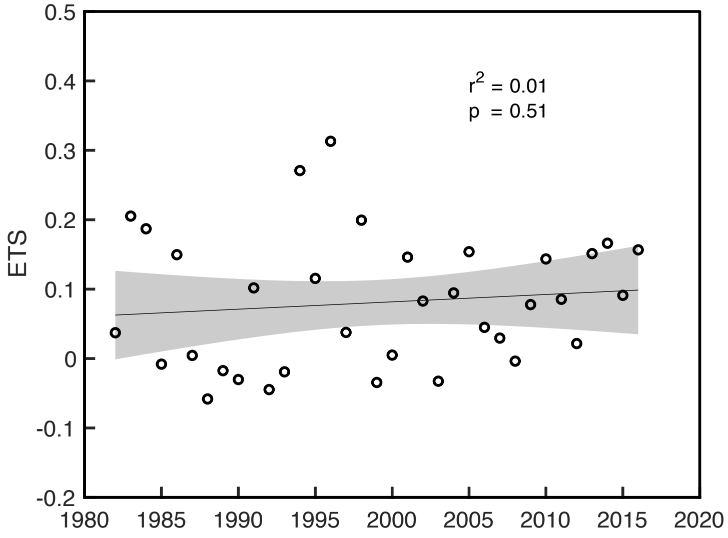

Figure S2. Temporal changes in ETS to evaluate whether predictive skill of bleaching events changes over time. There is no trend in ETS, suggesting that there are not substantial inaccuracies in the database during earlier years. Here, ETS is calculated using a 9-week DHW definition and excluding the 1 °C cutoff above the MMM. Black line and gray error bound indicates a linear fit with 95% confidence limits, which is not significant ($p > 0.05$).

Table S1. Corrections to site coordinates made to the original Hughes et al. (2018) bleaching database.

Site name	Original latitude	Original longitude	Corrected latitude	Corrected longitude	Notes
Australia, GBR Central	19.5°S	148.5°E	18.5°S	147.0°E	Original location very close to central/southern GBR boundary, changed to near middle of center GBR sector
Australia, GBR Southern	23.5°S	150.1°E	21.0°S	150.1°E	Original location too far south
Australia, Kimberly Coast	21.5°S	115.4°E	16.5°S	123.0°E	Original location in the Pilbara region, not in the Kimberley
Australia, Pilbara	19.5°S	119.9°E	21.5°S	115.4°E	Original location in the Kimberley region, not the Pilbara
Philippines, Central/Southern	9.4°N	120.0°E	12.0°N	123.0°E	Original location in Sulu Sea, not Philippines main islands
Vietnam, Con Dao Archipelago	14.3°N	109.3°E	8.7°N	106.6°E	Original coordinates in Vietnam, but >600 km away from the Con Dao Archipelago
Taiwan, Southern	23.7°N	121.0°E	22.3°N	120.5°N	Original location in center of Taiwan, not southern Taiwan coast
Madagascar, Southwest	20.5°S	46.5°E	24°S	43.5°E	Original location in center of Madagascar, not southwestern Madagascar
Sri Lanka	7.3°N	80°S	7.3°N	80°E	Original longitude coordinates listed as “80°S”, changed to “80°E”
Cook Islands	21.3°	159.8°W	21.3°S	159.8°W	Original latitude listed as “21.3°”, changed to “21.3°S”
Hawaii (main islands)	19.5°N	155.5°W	21.3°N	157.5°W	Original location is center of the Big Island of Hawaii, but most of the bleaching reports are from Oahu
Johnston Atoll (USA)	16.3°N	169.5°E	16.3°N	169.5°W	Original longitude in °E, but should be °W
Panama (Gulf of Panama)	8.5°N	79.1°E	8.5°N	79.1°W	Original longitude in °E, but should be °W

Table S2. Corrections to bleach events in the Hughes et al. (2018) bleaching database.

Site name	Change from	Change to	Notes
Australia, GBR Central & Australia, GBR Northern	1983 moderate	1982 severe	Oliver (1985): "Extensive bleaching of at least 25 species of hard coral and 2 species of soft coral was observed on reefs in the central and northern Great Barrier Reef during January to March 1982. Resultant mortality was high and it is estimated that more than 50% of living coral cover of some reefs died… Although the total number of reefs where bleaching was recorded in 1982 is fairly small (14), it includes almost every reef which was being regularly visited by Townsville scientists at the time." Fisk and Done (1985): "Mass bleaching of corals occurred on the Great Barrier Reef in early 1982…" See also Harriott (1985).
Galapagos	1987 severe	1987 moderate	Glynn et al. (2001): "Since the moderately strong ENSOs of 1987 and 1990-95 (Trenberth and Hoar, 1996) caused only moderate to light coral bleaching in Panama and the Galapagos Islands…" Podesta et al. (1997): "Moderate bleaching, affecting about 10% of all *Pocillopora* spp. colonies, was observed in the Galapagos Islands during the early months of 1987."
Saudi Arabia, Al Lith	2010 severe	2010 no bleaching	None of the literature cited in Hughes et al. (2018) actually shows (or even claims) that bleaching occurred in Al Lith during 2010. Additionally, coral skeletal cores show no signs of 2010 bleaching (DeCarlo et al., unpublished data).

Table S3. Changes in ETS and Bias associated with different bleaching predictors and responses, for the analysis with the Hughes et al. (2018) database without corrections to bleaching events as noted in Table S2.

**Conditions evaluated**	**Max ETS**	**ΔETS**	**Bias at max ETS**
Including 1 °C cutoff for DHW[a,b]	0.1483	-	0.9917
Excluding 1 °C cutoff for DHW[a,b,d]	0.1628	+0.0145	1.1767
DHW defined with 9-week window[a,c,d]	0.1674	+0.0046*	1.2733
Including El Niño threshold[a,c,d]	0.1964	+0.0290	0.8383
Including regional DHW thresholds[a,c,d]	0.2011	+0.0337	1.2467
Including El Niño and regional DHW thresholds[a,c,d]	0.2163	+0.0489	0.8283
Only "severe" bleaching events[c,d]	0.1883	+0.0209	1.4749
Maximum Hotspots[a]	0.1026	-0.0648	1.4417
Maximum Marine Heatwave index[a]	0.1251	-0.0423	0.9833

[a]*analysis of bleaching presence (including "moderate" and "severe") versus absence*
[b]*DHW defined with 12-week window*
[c]*DHW defined with 9-week window*
[d]*DHW defined without the 1° C cutoff*
**all subsequent ΔETS are relative to this value*
